# Case Study of an Atypical Presentation of a Large Undiagnosed Empyema in the Setting of Hyperglycemia

**DOI:** 10.7759/cureus.87599

**Published:** 2025-07-09

**Authors:** Cameron Bear, Jane Dow, Andrew Pham, Wasif Hafeez, Mohamed S Siddique

**Affiliations:** 1 Medical Education, Wayne State University School of Medicine, Detroit, USA; 2 Family Medicine, Detroit Medical Center Grand River Health Center, Detroit, USA; 3 Internal Medicine/ Infectious Disease, Detroit Medical Center Sinai-Grace Hospital, Detroit, USA; 4 Internal Medicine, Detroit Medical Center Sinai-Grace Hospital, Detroit, USA

**Keywords:** atypical presentation, community-acquired pneumonia, diabetic ketoacidosis, loculated empyema, streptococcus intermedius

## Abstract

A 58-year-old man with a history of type 2 diabetes mellitus was hospitalized for hyperglycemia. During a review of systems, the patient reported additional symptoms of mild, right-sided pleuritic chest pain. Further investigation revealed dullness to percussion with decreased breath sounds and slight tachycardia on physical exam. Additional workup revealed a 16 cm pleural effusion on CT, despite no fever, no leukocytosis, and mild clinical presentation. Thoracentesis findings revealed empyema, and laboratory analysis identified *Streptococcus intermedius*. Chest tubes were placed and antibiotics administered during a 15-day hospital stay until the patient requested to leave against medical advice.

No concerns of respiratory infection were indicated during the patient’s initial admission to the emergency department, nor during the patient’s previous hospitalization at a different facility one month prior. The patient remained afebrile (except for one recorded mild fever) with no leukocytosis for the entirety of the admission. This case study serves to increase awareness that even a large empyema can go undiagnosed when clinical presentation deviates from the expected and to inform physicians' response to improve patient health outcomes.

## Introduction

Empyema is an infection that results in the accumulation of pus in the pleural space [1]. This ailment is often a complication of either bacterial pneumonia or a paraneoplastic cause. It follows a three-stage progression: (1) exudative, (2) fibropurulent, and (3) organizing [1]. Initial symptomology of the exudative stage consists of fever, cough, chest pain, and dyspnea. During the fibropurulent stage, patients experience high fevers, purulent cough, malaise, and night sweats. If still untreated, the empyema will progress to the third stage in which patients exhibit cachexia, severe dyspnea, and exercise intolerance [1]. Per the American Association for Thoracic Surgery (AATS) Empyema Guidelines in 2017 by Shen et al., clinicians should be suspicious of empyema when their patient presents with signs and symptoms of either pneumonia or unexplained sepsis [2]. One of the main symptoms shared between these illnesses is the presence of a fever [1-2]. Many studies put great emphasis on the prevalence of fever in empyema. In fact, in one study analyzing 100 adults with empyema, fever was present in 95% of cases [3]. That said, the most agreed-upon prevalence of fever reiterated in literature is around approximately 73% [4]. Atypical presentations lacking these symptoms, though uncommon, usually present in immunocompromised and elderly individuals [5-6]. Initial evaluation to diagnose empyema is through a chest X-ray or CT thorax. Additionally, a lung pleural ultrasound can be utilized to both visualize the fluid and guide therapeutic/diagnostic tapping of the pleural space [2]. Treatment of empyema often consists of antibiotics and therapeutic removal of the pus collection. Depending on the duration and severity of the infection, patients may need serial thoracentesis or a video-assisted thoracoscopic surgery (VATS) procedure [2]. For these reasons, empyema treatment requires a team of different specialties, such as infectious disease, thoracic surgery, and pulmonology.

With this understanding of empyema in consideration, the presentation of empyema without fever and leukocytosis is an uncommon finding, particularly in an empyema of extensive size. Lack of presenting symptoms deters deduction of etiology, which informs appropriate, timely treatment, thus increasing the risk of complications and mortality [1]. The high morbidity and mortality associated with empyema necessitate deeper exploration into the causes of atypical presentations to improve recognition and timely diagnosis in the future. The objective of this case study is to present a case of a large empyema that diverged from the common presentation, in the context of an alternate chief complaint, to empower practitioners when faced with atypical presentations to pursue workups that may deviate from standard conventions.

## Case presentation

A 58-year-old man with a past medical history of type 2 diabetes mellitus, hypertension, and hyperlipidemia presented to the emergency department (ED) for concerns of diabetic ketoacidosis (DKA) after a high blood glucose reading. The patient admitted to non-compliance with medication and inconsistent glucose monitoring. The patient was treated for DKA one month prior to this admission at a different facility and discharged without adequate medical follow-up. While taking the history, the patient noted right-sided chest discomfort, which he attributed to an altercation two to three weeks ago. 

On presentation, the patient was in no acute distress, sitting comfortably, with stable vitals aside from mild tachycardia as depicted in Table 1. He noted associated symptoms of polyphagia, polydipsia, polyuria, and chronic peripheral neuropathy controlled with gabapentin. In terms of social history, he smoked a half pack of cigarettes daily for 40 years, denied alcohol use, and denied other recreational drug use. Blood glucose was 691 mg/dL without anion gap, mild elevation of beta-hydroxybutyrate, and urine positive for glucose but negative for ketones. 

**Table 1:** Vitals on admission to ED demonstrating tachycardia amongst otherwise normal vitals; notably afebrile. bpm: beats per minute; mmHg: millimeters of mercury; gm/dL: grams per deciliter; SpO2: saturation of peripheral oxygen; C: Celsius

Vitals on Admission	Result	Reference Range
Blood pressure (mmHg)	109/72	90-140/55-90
Heart rate (bpm)	119	60-100
Respiratory rate (breaths per minute)	18	14-20
SpO2 (%)	96%	Normal: 92-100%; critical low: 88%; critical high: 100%
Temperature (C°)	37.5	Normal: 35.7-37.5; critical low: 35.3; critical high: 38.4

Initial blood workup was otherwise unremarkable; complete blood count (CBC) revealed mild anemia and notably no leukocytosis or left shift as seen in Table 2. Basic metabolic panel (BMP) showed pseudo-hyponatremia, and troponin was 8 ng/L, and the electrocardiogram was normal with no ischemia.

**Table 2:** CBC on admission to ED demonstrating mild anemia; notably no leukocytosis. K/CUMM: thousands per cubic millimeter; gm/dL: grams per deciliter; CBC: complete blood count; WBC: white blood cells

CBC on Admission	Result	Reference Range
WBC (K/CUMM)	9.1	3.5-10.6
Hemoglobin (gm/dL)	12.4	13.3-17.1
Hematocrit (%)	38.2	38.9-49.7
Platelets (K/CUMM)	436	150-450

**Figure 1:**
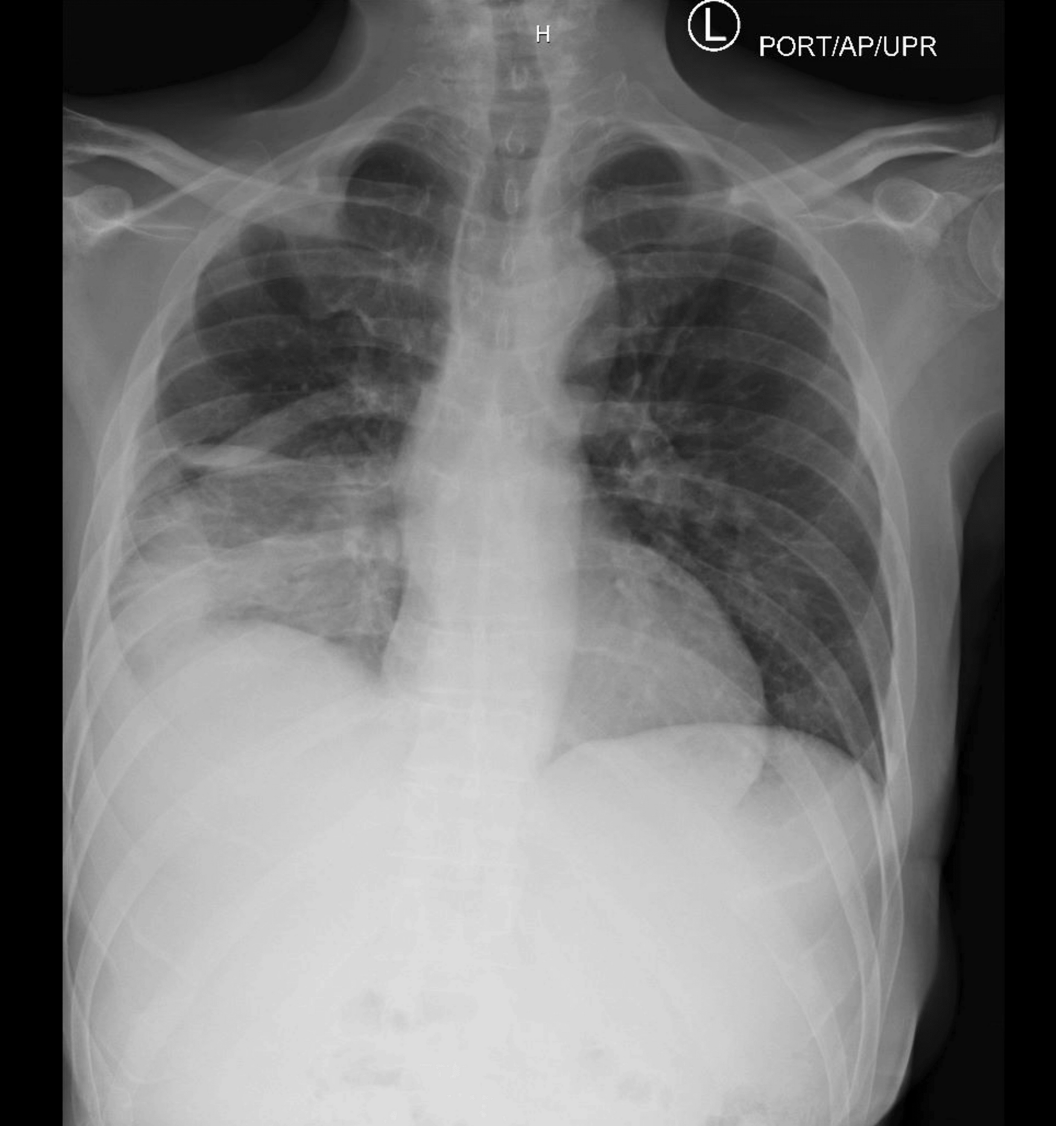
Initial chest x-ray. Impression read as right lower lobe pneumonia with small right pleural effusion.

On further review of systems with the medicine team, he denied any history of fever, chills, hemoptysis, loss of appetite, abdominal pain, or previous lung infection. He did, however, confirm weight loss of 10 pounds over two months. On physical examination, there was dullness to percussion and decreased breath sounds in the right lower lobe. On hospital Day 1, a chest X-ray, depicted in Figure 1, revealed consolidation and a right pleural effusion. The ED administered 10 units of insulin, and the patient was started on azithromycin 500 mg and ceftriaxone 2 g for community-acquired pneumonia. The patient was admitted for further management of uncontrolled diabetes mellitus and pneumonia. 

**Figure 2:**
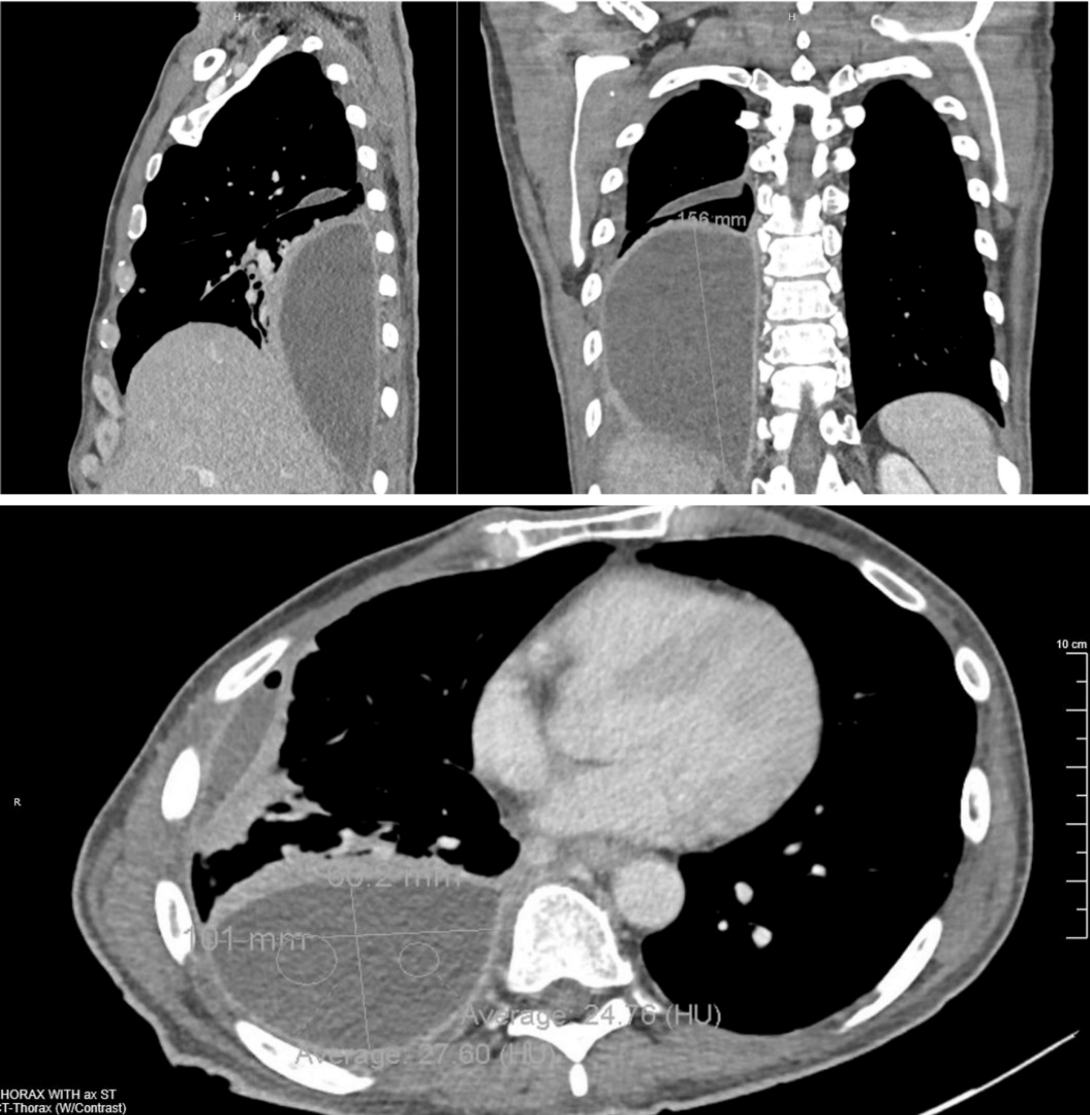
CT revealing empyema encompassing the right lower pleural space CT: computed tomography

A follow-up CT, depicted in Figure 2, demonstrated a large loculated posterior pleural effusion in the hemithorax adjacent to the right lower lung measuring 16 cm in cephalocaudal direction and a second loculated fluid collection adjacent to the right middle lobe measuring 5 cm in cephalocaudal direction. For comparison, empyema > 2.5 cm on CT is considered a moderate-to-large effusion that requires intervention [7-8]. Infectious disease (ID) was consulted, and the following recommendations were implemented for empiric treatment: azithromycin 500 mg was switched for doxycycline 100mg twice a day, and ceftriaxone 2 g was continued with further recommendations pending pleurocentesis. On hospital Day 4, due to minimal clinical improvement, ID recommended vancomycin 1000 mg for staph coverage.

Interventional radiology placed two chest tubes and performed pleurocentesis on hospital Day 4. Fluid samples were obtained; the results of which are recorded in Table 3. Once cultures resulted in *Streptococcus intermedius*, vancomycin was discontinued, and the patient continued with ceftriaxone 2 g. In addition to drainage, tube placement, and antibiotics, the patient received three rounds of tissue plasminogen activator (tPA) and dornase during the course of his hospital stay.

**Table 3:** Results of the pleurocentesis. Glucose is relatively high, though lower than serum glucose. Elevated LDH and protein levels are suggestive of an exudative effusion. Lipase is mildly elevated but not markedly so. Nucleated cell count is significantly elevated. LD: lactate dehydrogenase; RBC: red blood cells; PMN: polymorphonuclear leukocytes; /CUMM: per cubic millimeter; mg/dL: milligrams per deciliter; U/L: units per liter; gm/dL: grams per deciliter

Pleurocentesis Results	Result	Reference Range
Glucose (mg/dL)	156	75-105
LD (U/L)	>6000	The reference range and other method performance specifications have not been established for this body fluid. The test result must be integrated into the clinical context for interpretation.
Lipase (U/L)	48	The reference range and other method performance specifications have not been established for this body fluid. The test result must be integrated into the clinical context for interpretation.
Protein (gm/dL)	2.9	The reference range and other method performance specifications have not been established for this body fluid. The test result must be integrated into the clinical context for interpretation.
Appearance	Turbid	
Color	Pink	
RBC (/CUMM)	130,320	Fluid RBC (0-1/CUMM)
Nucleated cells (/CUMM)	489,280	Fluid nucleated cells (0-200/CUMM)
Neutrophil %	Cells degenerated for valid differential	
Fluid culture	Gram stain: Numerous gram-positive Cocci. Numerous PMNs’ seen. Results: *Streptococcus intermedius.*	
Final cytology interpretation	Specimen: Pleural fluid with cell block adequacy; satisfactory for evaluation. Interpretation: negative for malignant cells. Abundant neutrophils noted.	

Over a 15-day hospital stay, the patient remained vitally stable; he was afebrile apart from one mild fever of 38.2 °C on hospital Day 3, and white blood cell count remained within normal limits. Additional labs for tuberculous mycobacteria, legionella, aspergillus, and HIV were all negative. The chest tubes drained 1.37 L prior to removal per patient request to leave against medical advice (AMA). Counseling was provided on leaving AMA, the drains were removed, and the patient was to be discharged on a four-week course of Augmentin 875mg twice daily. The last chest X-ray taken on the day the patient left the hospital noted a stable chest with strandy right lower lobe opacities consistent with subsegmental atelectasis and possible small right pleural effusion. 

In summary, this patient presented with symptoms of uncontrolled diabetes mellitus and right-sided pleuritic chest discomfort. Lack of fever, no leukocytosis, and normal physical exam deterred further infectious workup. That said, persistent clinical practice, including consistent and targeted physical exam, communication with the patient, and advocacy for an extensive work-up, led to the treatment of a massive, undiagnosed empyema.

## Discussion

This case is remarkable given its divergence from the classical presentation of empyema as represented by Table 4, particularly considering the impressive size of the loculation. 

**Table 4:** The divergence in presentation of the present case from a typical presentation of empyema

Feature	Typical empyema [1]	Present case
Fever	Present in >90% of cases	Absent, one transient fever (38.2°C) on Day 3
Leukocytosis	Common, often with left shift	WBC normal (9.1), no left shift
Pleuritic chest pain	Common	Mild, right-sided, patient-attributed to trauma
Dyspnea	Often severe	Only mild exertional
Imaging findings	Often show consolidation + effusion	CT: large (16 cm) loculated effusion
Organism	Strep pneumoniae, anaerobes, S. aureus	Streptococcus intermedius (oral flora)
Risk Factors	Pneumonia, aspiration, immunocompromise	Type 2 diabetes, smoking (20 pack-year)
Outcome	Often prolonged hospitalization	Discharged AMA after 15 days

Typically, empyema presents with specific symptoms of cough, fever, pleuritic chest pain, dyspnea, sputum production, and in extreme cases, septic features. Furthermore, labs indicate leukocytosis, left shift, and elevated C-reactive protein [1]. Patients with empyema tend to present as sickly and possess a significant comorbidity or immunocompromised state [9]. 

This patient, with an unrelated chief complaint, only demonstrated mild pleuritic chest pain and occasional exertional dyspnea. These findings were confirmed on review of systems rather than from direct concern of the patient. 

Of note, studies have found that the size of an empyema correlates with the severity of systemic symptoms [1]. As such, a 16 cm loculation with such mild symptoms is unusual. 

According to the AATS by Shen et al., most individuals experience symptoms two weeks prior to hospitalization [2]. Since this patient did not express obvious symptoms, it is challenging to ascertain a timeline and subsequently an etiology, which usually aids in targeted treatment. Notably, during his admission for DKA a month prior, there was no reported pneumonia workup, and the patient was not prescribed antibiotics. It is unclear whether the empyema existed during the previous hospitalization. 

Early diagnosis and treatment of empyema are critical for reducing morbidity and mortality. Non-malignant pleural effusion has a 25-57% mortality within the year [10]. Empyema is associated with prolonged hospital stays, on average 19 days, increasing the risk for hospital-acquired infections [1]. In patients with delayed diagnosis, greater than 7 days, hospital stays averaged 28 days, with increased need for surgery and higher rates of complications such as pleural fibrosis, bronchopleural fistula, sepsis, and respiratory failure [11-13]. Therefore, optimized diagnosis and treatment are critical; however, this is challenging when the clinical picture is unclear. This is of further importance with larger loculated empyema. A study by Chung et al. found that larger effusions were associated with a higher risk of treatment failure due to higher levels of vascular endothelial growth factor and interleukin-8 [14]. Therefore, larger effusions pose a greater risk of complications. 

It is helpful, for forming a differential and expediting effective treatment, to consider known cases of empyema lacking fever and leukocytosis. One cause includes spontaneous bacterial empyema (SBEM) [15]. That said, most cases are associated with cirrhosis and ascites. The infection spreads contiguously in the accumulated fluid through the diaphragm, leading to the development of empyema. Following a similar mechanism, fluid-overloaded states, such as end-stage renal disease (ESRD), in immunocompromised patients can result in empyema formation from infected fluid [15]. 

This patient did not have cirrhosis or ESRD, and workup for an immunocompromised state was negative except for type 2 diabetes. It is worth considering the role of diabetes in this atypical presentation. Research has demonstrated that diabetes affects the innate and adaptive immune responses [16]. That said, uncontrolled hyperglycemia is primarily linked to higher levels of proinflammatory cytokines such as TNF-α and IL-6, which lead to an increase in fever and leukocytosis. Therefore, one would expect to present with a more pronounced clinical presentation [17]. It is possible that the hyperglycemic-induced dysregulation of the immune system could have the potential to blunt systemic effects to the degree of facilitating an atypical clinical presentation, and as such, cannot be ruled out as a potential contributing factor in this case.

A more pertinent risk factor, however, includes the patient’s substantial smoking history (20 pack years). According to AATS Consensus Guidelines, empyema without fever or leukocytosis can be seen in anaerobic pleural infections amongst individuals with poor dental hygiene and at increased risk of aspiration [2]. Extensive use of tobacco increases the risk of community-acquired pneumonia via aspiration [18]. *Streptococcus intermedius*, a normal component of the oral cavity microbiota, is a frequent culprit in community-acquired pleural empyema [19]. Another study found a case in which a 40-year-old man who smoked 10 cigarettes a day since the age of 18, with no other risk factors, presented with empyema without fever or leukocytosis. The bacteria in this case were *Fusobacterium nucleatum;* however, the shared social history and atypical presentation indicate a cause for future research into the impact of cigarettes [20].

Given this patient's smoking history, lack of fluid overload, and no definitive immunocompromised status, it is possible that community-acquired pneumonia due to aspiration of *Streptococcus intermedius* is responsible for the empyema (however, potential effects of diabetes on the immune system, though unlikely as discussed previously, can’t be entirely ruled out). Aspiration of anaerobic bacteria has been responsible for previous occurrences of empyema without fever or leukocytosis. Pleural effusion can develop between two to six weeks. This suggests the possibility that this empyema existed during the previous admission, and atypical presentation masked and delayed early diagnosis and intervention. 

Limitations of this report include the absence of imaging from the prior hospitalization and incomplete outcome data due to the patient’s early discharge against medical advice. Nevertheless, the findings reinforce the need for persistent clinical suspicion and the potential value of point-of-care imaging in uncovering occult infections when typical signs are absent.

## Conclusions

This case underscores the diagnostic challenge posed by atypical presentations of empyema, particularly in patients with underlying risk factors such as diabetes mellitus and an extensive history of smoking. Despite a massive 16 cm loculated pleural effusion, this patient remained largely asymptomatic, afebrile, and without leukocytosis, features that typically prompt earlier investigation and intervention.

This case demonstrates that even large, advanced-stage empyemas may present subtly and emphasizes the importance of comprehensive physical examination, thoughtful review of systems, and maintaining a broad differential diagnosis. It also raises questions about how chronic hyperglycemia and tobacco exposure may alter immune responses and blunt systemic manifestations of infection. Clinicians should be particularly vigilant in patients with aspiration risk or poor glycemic control, as early recognition and drainage of empyema are critical to improving outcomes and reducing morbidity.
